# Li-ion half-cells studied *operando* during cycling by small-angle neutron scattering

**DOI:** 10.1107/S160057671901714X

**Published:** 2020-02-01

**Authors:** Johannes Hattendorff, Stefan Seidlmayer, Hubert A. Gasteiger, Ralph Gilles

**Affiliations:** a Chair of Technical Electrochemistry, Department of Chemistry and Catalysis Research Center, Technische Universität München, Lichtenbergstrasse 4, Garching 85748, Germany; b Heinz Maier-Leibnitz Zentrum (MLZ), Technische Universität München, Lichtenbergstrasse 1, Garching 85748, Germany

**Keywords:** small-angle neutron scattering, SANS, batteries, lithium, graphite, lithiation process

## Abstract

Small-angle neutron scattering (SANS) was recently applied to the *in situ* and *operando* study of the charge/discharge process in Li-ion battery full-cells based on a pouch cell design. Here, this work is continued in a half-cell with a graphite electrode cycled *versus* a metallic lithium counter electrode, in a study conducted on the SANS-1 instrument of the neutron source FRM II at the Heinz Maier-Leibnitz Zentrum in Garching, Germany. It is confirmed that the SANS integrated intensity signal varies as a function of graphite lithiation, and this variation can be explained by changes in the squared difference in scattering length density between graphite and the electrolyte. The scattering contrast change upon graphite lithiation/delithiation calculated from a multi-phase neutron scattering model is in good agreement with the experimentally measured values. Due to the finite coherence length, the observed SANS contrast, which mostly stems from scattering between the (lithiated) graphite and the electrolyte phase, contains local information on the mesoscopic scale, which allows the development of lithiated phases in the graphite to be followed. The shape of the SANS signal curve can be explained by a core–shell model with step-wise (de)lithiation from the surface. Here, for the first time, X-ray diffraction, SANS and theory are combined to give a full picture of graphite lithiation in a half-cell. The goal of this contribution is to confirm the correlation between the integrated SANS data obtained during *operando* measurements of an Li-ion half-cell and the electrochemical processes of lithiation/delithiation in micro-scaled graphite particles. For a deeper understanding of this correlation, modelling and experimental data for SANS and results from X-ray diffraction were taken into account.

## Abstract 2

Lithium-ion half-cells are studied *in situ* and *operando* by small-angle neutron scattering. The variation in the signal can be explained by the lithiation of graphite in the sample.

## 1. Introduction   

The increased research interest in Li-ion batteries has triggered the development of new methods to study the detailed processes occurring inside a battery cell. Small-angle neutron scattering (SANS) has recently been used for studying battery materials *in situ* and *operando* in functional batteries (Sacci *et al.*, 2015 ▸; Bridges *et al.*, 2012 ▸; Zhu *et al.*, 2019 ▸; Risse *et al.*, 2019 ▸). The SANS technique gives information on the phase distribution on a mesoscopic scale and is thus complementary to neutron diffraction, which gives information regarding (global) phase existence and properties on an atomic scale. Both methods probe a sample volume typically of the order of 1 cm^3^ in order to obtain results with sufficient statistics. In earlier work (Seidlmayer *et al.*, 2015 ▸), we performed the first *in situ* scattering experiments with a full-cell Li-ion battery composed of a graphite anode and an NMC111 (LiNi_0.33_Mn_0.33_Co_0.33_O_2_) cathode. It was shown that the graphite component dominates the scattering signal of an NMC111/graphite full-cell. A core–shell model was proposed, describing the scattering contrast from the near-surface region of the graphite active material particles (here referred to as shell) and the surrounding electrolyte phase, with a local resolution based on the SANS signal coherence length. In this follow-up work, our aim is to answer several questions which remained unresolved in that report. Could the signal have been influenced by factors other than the graphite material, *e.g.* the cathode active material? Can we assume that the electrode represents a sufficiently homogeneous system in the SANS measurements, so that SANS is really representative of any given graphite particle in the cell? Moreover, is the postulated quadratic dependence of the integrated intensity on the squared difference of scattering length density valid? In this article, we combine X-ray diffraction (XRD) and SANS in order to view the lithiation of graphite from a new perspective. A new theoretical model supports the experimental results.

Several SANS studies of carbon-based anode active mater­ials for Li-ion batteries have been conducted in the past. For example, Nagao *et al.* (2006 ▸) used *ex situ* SANS and other techniques to study hard carbon anode active materials. More recently, Sacci *et al.* (2015 ▸) employed *in situ* SANS to study the solid–electrolyte interface (SEI) formation on graphite, and earlier Bridges *et al.* (2012 ▸) used the same approach to examine SEI evolution in nano-sized pores in hard carbon anode active materials by following changes in the scattering length density. In these studies, the authors implicitly assumed that the scattering signal from the contributing materials scales with the squared difference in scattering length density, weighted by the material volume fraction, a relationship which, to the best of our knowledge, is derived for the first time in the present work (see Appendix *A* [App appa]). The first *operando* SANS data recorded during the cycling of an Li/graphite half-cell were reported by Wang *et al.* (2012 ▸). For graphite, the coexistence of various Li_*x*_C phases or stages has long been determined by *in situ* XRD (Dahn, 1991 ▸; Dahn *et al.*, 1990 ▸). To explain the lithiation process of graphite, Heß & Novak (2013 ▸) considered the graphite particles as homogeneous and proposed that the lithiation of a graphite particle for each given stage proceeds from the outside to the centre of the graphite particle. The existence of such a lithiation front, which always separates two distinct phases, has also been supported by numerical simulations with an advanced lithium solid-phase mass-transport model (Bohn *et al.*, 2013 ▸). This suggests that a stable front of (lithiated) graphite phases can proceed through a graphite particle even at high charge rates (at C-rates of *ca* 0.7 C; C-rate is understood as current over capacity, as usual). This is often called the ‘shrinking core model’, but it may not necessarily be valid with different carbon-based anode materials (*e.g.* multi-domain mesocarbon microbeads) or at different rates, for which other lithiation patterns on the single-particle level have also been reported (Harris *et al.*, 2012 ▸). Recently, multiple coexistent phases in lithiated graphite have been reported in neutron diffraction experiments as well, described as a phase inhomogeneity or graphite phase gradient (Zinth *et al.*, 2017 ▸).

## 2. Experimental   

**Figure 1:**
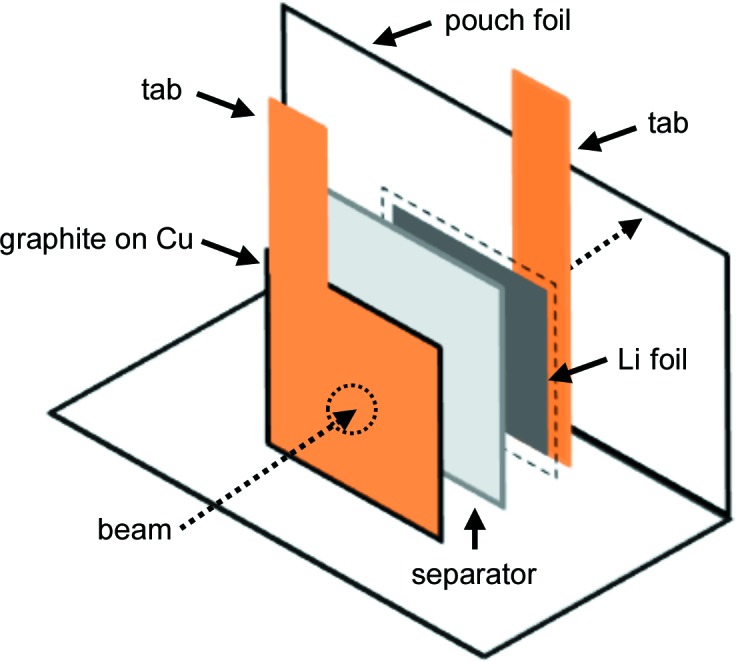
The Li/graphite pouch half-cell setup and neutron beam direction. The dimensions of the graphite electrode on the copper current collector are 2.6 × 2.6 cm (= 6.76 cm^2^). Those of the Li foil are 2.8 × 2.8 cm (= 7.84 cm^2^). The 450 µm thick Li foil is contacted by a copper tab on the side of the Li foil outside the neutron beam with a width of *ca* 1 cm.

Li/graphite pouch half-cells were built in an argon-filled glove box. The half-cells consisted of a graphite-coated copper foil (SGL Carbon T157) as the working electrode and a metallic Li foil of 450 µm thickness (Rockwood Lithium) as the counter electrode. The latter was contacted electronically by a copper current-collector tab at the side of the electrode, located outside the area through which the *ca* 1 × 1 cm sized neutron beam passes (for dimensions see the caption of Fig. 1 ▸). The potato-shaped synthetic graphite particles had a mean diameter of 22 µm as determined by laser diffraction particle sizing (Retsch-Horiba LA-950). The electrodes were separated by a polyolefine separator (Celgard C2013) which was soaked in an electrolyte composed of ethyl­ene carbonate/ethyl methyl carbonate (3:7 wt%) with 1 *M* LiPF_6_ and 2 wt% vinyl­ene carbonate (BASF LP572). The theoretical areal capacitance of the graphite electrode was 1.63 mA h cm^−2^ (based on a theoretical capacity of 360 mA h g^−1^). The thickness of the copper foils used was 12 µm (MTI Corporation) and the Al foil (MTI Corporation) in the pouch casing was 40 µm thick. The overall thickness of the Li/graphite cell used in this experiment was ∼1 mm.

For comparative measurements, a symmetric Li/Li cell was built, in which the 450 µm thick Li foils were also supported on a copper current-collector frame with a central window of the same dimensions as for the Li/graphite cell. The smaller electrode in each of the two cells was 6.76 cm^2^ in all cases. The cells were cycled with a potentiostat (Biologic, France). Prior to the SANS experiments, the Li/graphite half-cell underwent two formation cycles at C/10 (≡ 0.16 mA cm^−2^) and two cycles at C/5 (≡ 0.32 mA cm^−2^) between 0.01 and 1.5 V. The Li/Li cell did not undergo any formation prior to the SANS experiments.

All *operando* SANS experiments were undertaken on the SANS-1 instrument at the FRM II of the Heinz Maier-Leibnitz Zentrum (MLZ) in Garching, Germany (Gilles *et al.*, 2006 ▸; Mühlbauer *et al.*, 2016 ▸; Heinemann & Mühlbauer, 2015 ▸). Measurements were performed with a neutron wavelength of 6 Å and a sample-to-detector distance of 8 m (the same value was chosen for the collimation) to cover a medium *q* range of 0.068–0.934 nm^−1^ [*q* = (4π/λ) sinθ, where θ is half the scattering angle and λ is the wavelength of the incident beam]. Accumulated signals from this *q* range were saved every 3 min to a data file and data reduction, including calibration, was performed with the *BerSANS* software (Keiderling, 2002 ▸). In general, the signal was normalized with respect to the detector dead time and it was corrected for the fluctuating beam intensity, which was measured at the beam entrance, *i.e.* normalization was done with respect to the ‘empty’ beam.

In the *operando* SANS experiment with the Li/graphite cell, the preformed graphite electrode was lithiated from 1.5 to 0.01 V with a constant current (CC) at a rate of C/5, followed by a constant voltage phase (CV) with a cutoff current corresponding to C/10. Subsequently, the graphite electrode was delithiated at a constant current corresponding to C/5. Finally, after a short period in OCV (open circuit voltage) condition, the graphite was lithiated (CC-CV) and delithiated (CC) with C/2 in order to examine the SANS signal evolution at higher rates. This last cycle could not be fully completed due to limited beam time. After the SANS experiment, the Li/graphite half-cell was taken to an X-ray diffractometer (Empyrean, PANalytical, Almelo, Netherlands) at the Mater­ials Science Laboratory of the MLZ, equipped with an Mo X-ray tube (λ = 0.71 nm, *K*α_1_ and *K*α_2_) operated at 40 mA and 55 kV, where it was cycled at C/5 as in the SANS experiment while collecting *operando* XRD patterns in transmission mode every 6 min.

The symmetric Li/Li cell underwent no formation and was brought directly to the SANS instrument and cycled at a current density corresponding to that of C/5 for the Li/graphite half-cell (*i.e.* at 0.32 mA cm^−2^). The cell was cycled first for 2 h in one direction and then for a further 2 h in the other direction. The Li transfer in the Li/Li cell happened at an overvoltage of ∼100 mV.

## 3. Results and discussion   

### 3.1. XRD measurements   

**Figure 2:**
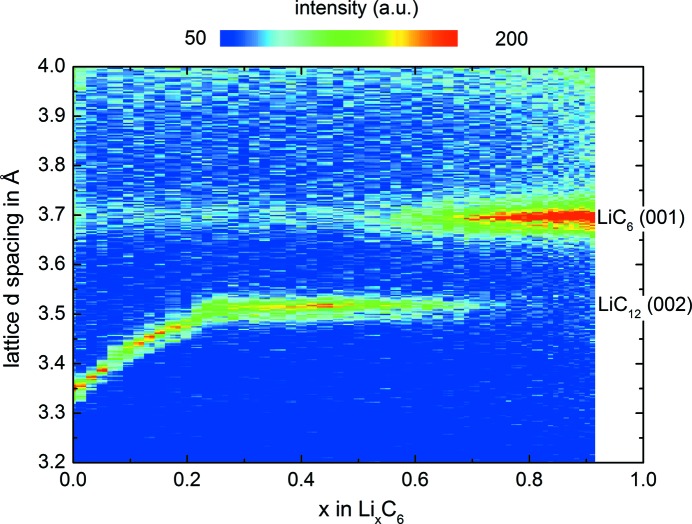
An *operando* X-ray diffractogram recorded from the Li/graphite half-cell cycled at C/5, showing the presence of well defined lithiation stages, which indicates good homogeneity across the electrode.

Fig. 2 ▸ shows an *operando* X-ray diffractogram of the Li/graphite half-cell which was measured in a setup like that shown in Fig. 1 ▸. During cycling at the relatively low C-rate of C/5 there are clear transitions from the lower lithiated phases to LiC_12_ and then to LiC_6_. Thus, at higher lithiation (*x* > 0.2) LiC_12_ and LiC_6_ are the two phases present in the sample. The data in Fig. 2 ▸ show the range of *d* spacings around the (00*l*) reflections of graphite and the lithiated graphite phases. At the beginning of the lithiation process (from left to right), a non-continuous gradual shift of the 002 reflection of graphite with a lattice spacing of *d* ≃ 3.35 Å towards higher values is observable. A first discontinuity appears around *d* ≃ 3.43 Å (at *x* ≃ 0.1). A second discontinuity is visible at *d* ≃ 3.50 Å (at *x* ≃ 0.2), shortly before the lattice spacing of LiC_12_ is reached at *d* ≃ 3.53 Å and the 002 reflection of LiC_12_ appears. With increasing degree of lithiation, the 001 reflection of LiC_6_ gradually starts to appear at *d* ≃ 3.70 Å (from *x* ≃ 0.5) while the LiC_12_ reflection gradually disappears until full lithiation is achieved.

While the XRD data provide averaged phase distribution information which is consistent with the literature (Dahn, 1991 ▸; Senyshyn *et al.*, 2013 ▸; Dahn *et al.*, 1990 ▸), they provide no insight into the spatial distribution of the various phases on a particle-scale level. We did not observe the occurrence of more than two coexisting graphite stages, as observed, *e.g.* by Wilhelm *et al.* (2018 ▸) at low temperatures. Therefore, we assume that the lithiation within the graphite particles, as well as that within the entire electrode, is homogeneous. However, the detection limit for observing phase fractions in our XRD experiment is limited and phases with a small volume share might not have enough scattering power to be seen.

### 3.2. SANS measurements   

**Figure 3:**
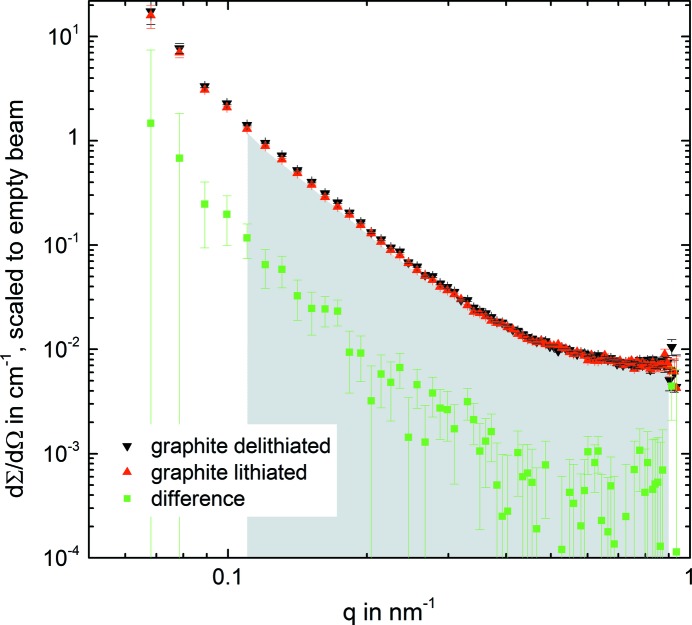
The *operando* determined macroscopic scattering cross section dΣ/dΩ versus *q* for fully delithiated graphite (black symbols) and for the fully lithiated LiC_6_ phase (red symbols) measured in the Li/graphite half-cell. The green symbols represent the difference between the SANS signal from fully delithiated graphite and that from the LiC_6_ phase. The SANS cross sections shown here were corrected for detector dead time and beam intensity. Error bars are calculated from the detector and beam errors and indicate the overall SANS data point error. The integrated intensity value Γ described in the text was determined by integration over the grey shaded area.

The same cell was measured in different lithiation states in an *operando* SANS experiment in order to provide mesoscopic information. Fig. 3 ▸ shows the scattering, *i.e.* the macroscopic scattering cross section dΣ/dΩ as a function of the wavevector *q* for the Li/graphite half-cell. The figure shows an exponential decrease in the macroscopic scattering cross section when going to larger *q* values, which then levels off to a constant background at *ca* 0.0075 cm^−1^. The exponential drop follows a *q* ^−*m*^ law, where the exponent *m* can be determined to be *m* = 3.8 (fit of all data points to *c*_0_ + *c*_1_ *q* ^−*m*^, where *c*_0_ and *c*_1_ are constants) using the software *SASfit* (Breßler *et al.*, 2015 ▸). This is very close to the classical Porod scattering with *m* = 4. Interestingly, there is a significant difference in the SANS signal between the lithiated and delithiated graphite states. This is clearly discernible in the difference curve in Fig. 3 ▸ (green symbols), where the SANS signal in the half-cell from the fully lithiated graphite is always below the one from the lithium-free graphite.

In order to better compare the difference between the various graphite phases during cycling, we can define an integral measure of the SANS signal which combines all data points into one value. We thus determine the integrated intensity Γ, which we obtain by integrating the normalized scattering cross section dΣ/dΩ over the *q* range highlighted in grey in Fig. 3 ▸ (here 0.11–0.89 nm^−1^). The integration range was limited in order to exclude data points with a large error.
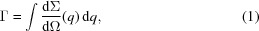
integrated in this work from 0.11 to 0.89 nm^−1^.

**Figure 4:**
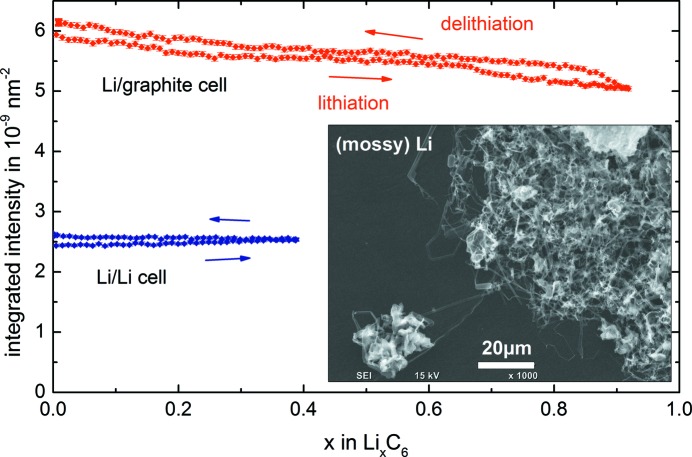
The integrated intensity Γ of the *operando* SANS signal [defined by equation (1)] obtained from the Li/graphite half-cell (red points) and the Li/Li cell (blue points) during one cycle at a current density of 0.32 mA cm^−2^ (corresponding to C/5 for the Li/graphite half-cell). The inset shows a scanning electron microscopy image of the pristine Li foil (dark areas) and Li dendrites grown on top of it.

The upper line (red) in Fig. 4 ▸ shows how the integrated intensity Γ of the SANS signal for the Li/graphite half-cell varies continuously and reversibly during a full lithiation and delithiation cycle at C/5. For comparison, the evolution of the SANS signal of the symmetric Li/Li cell cycled at the same current density is shown on the same axis, based on the transferred charge during cycling (2 h in each direction for the Li/Li cell, instead of *ca* 5 h in the case of the Li/graphite half-cell). The maximum variation in the integrated intensity Γ [see equation (1)] for the Li/graphite half-cell between *x* = 0 (fully delithiated) and *x* = 1 (fully lithiated) is 1.2 × 10^−9^ nm^−2^ and thus much higher than the variation observed for the Li/Li cell, which would only amount to 0.2 × 10^−9^ nm^−2^ when the slope of the measured data is extrapolated to *x* = 1. Note also that the integrated intensity Γ of the Li/graphite half-cell is reversible, whereas that of the Li/Li cell increases monotonically, and the absolute level of the integrated intensity Γ from the Li/graphite cell is significantly higher.

Upon closer inspection of Fig. 4 ▸ it is apparent that the integrated intensity Γ does not return to the same value after one cycle, which we believe is due to the following phenomenon. In a symmetrical Li/Li cell made from two fresh Li foils, the following surface modification occurs upon cycling: both electrodes start with a perfectly flat Li surface (dark-grey area in the inset of Fig. 4 ▸), but during cycling a micro-dendritic or mossy Li structure (lighter-grey structures) grows on the Li foil onto which lithium is being deposited, *i.e.* over the course of one cycle, the formation of lithium dendrite and mossy lithium can be observed on both Li foils of the Li/Li cell, consistent with the literature (Wandt *et al.*, 2015 ▸; Steiger *et al.*, 2014 ▸). Since these dendrites are of the order of micrometres, we can surely reject any direct visibility in the SANS signal. However, the growth of mossy Li results in an increase in surface area which can explain the continuous increase in the integrated intensity of the SANS signal of the Li/Li cell over the cycle shown in Fig. 4 ▸ (blue line). We therefore believe that the contribution to Γ produced by the surface area increase of the lithium electrode is responsible for the increase in Γ after one cycle (*i.e.* at *x* = 0) observed for the Li/graphite cell (red line). Nevertheless, owing to the overall very high integrated intensity for the Li/graphite cell, this signal contribution from the Li counter electrode is rather minor and the graphite electrode contributes the most to the SANS signal, which will be discussed below.

### 3.3. Detailed examination of the *operando* Li/graphite cell data   

**Figure 5:**
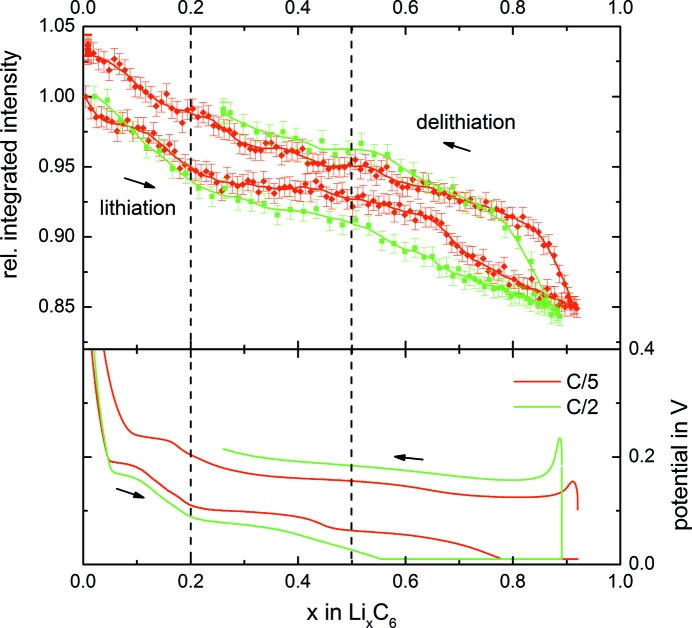
The integrated intensity Γ of the *operando* SANS signal [defined by equation (1)], normalized to the initial value at *x* = 0 (upper panel), and the cell potential (lower panel) of an Li/graphite half-cell *versus* the degree of lithiation (*x*) for rates of C/5 (red) and C/2 (green). The dashed vertical lines indicate the onset of LiC_12_ and LiC_6_ formation from XRD during charge.

The upper panel of Fig. 5 ▸ shows how the *operando* measured SANS integrated intensity varies with the degree of lithiation of the graphite electrode for a C-rate of C/5 (red line/symbols) and for a rate of C/2 (green line/symbols). Here, the integrated intensities of the SANS signals are normalized to the first value at *x* = 0 and the estimated error (see *Experimental*, Section 2) is given by the error bars; the lines represent a smoothed Savitzky–Golay fit (Savitzky & Golay, 1964 ▸) based on third-order polynomials. For comparison, the curves of potential *versus* degree of lithiation (*x*) are shown in the lower panel, exhibiting clearly visible plateaus for the various lithiation and delithiation stages of graphite. The dashed vertical lines mark the beginning of the phase transitions to LiC_12_ at *x* ≃ 0.2 and to LiC_6_ at *x* ≃ 0.5, which were observed in the above *in situ* XRD measurements (see Fig. 2 ▸). For the higher rate of C/2, increasing overpotentials make it more difficult to identify the plateau-like transitions between these phases.

During lithiation both integrated intensity curves drop by roughly 15% and feature an S shape with a lower slope in the middle section. The delithiation curve rises quickly at the beginning and then more slowly until the pure delithiated graphite is obtained again at an absolute level which is slightly above its initial value (see the above discussion of this phenomenon), amounting to a total scattering intensity rise of 19%. This clearly demonstrates that the integrated intensity of the SANS signal depends on both the state of charge (SOC) of the graphite electrode and on the charge/discharge direction. In the following we will relate this signal variation to the change in scattering length density during cycling.

## 4. Comparison of measured and theoretically predicted SANS signals   

**Figure 6:**
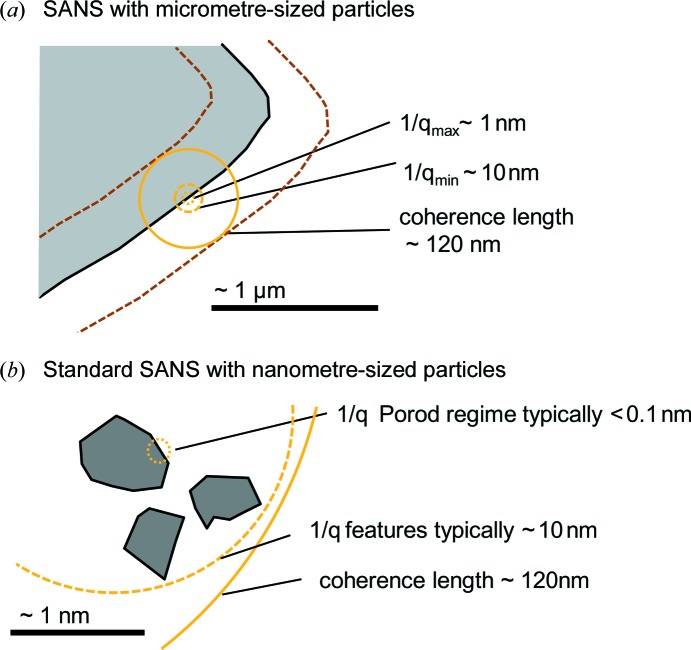
An illustration of the relevant length scales for SANS when conducted with (*a*) large micrometre-sized graphite particles (*i.e.* of the size of most battery active materials) or with (*b*) particles in the nanometre size scale, as is the case for most SANS applications. For the micrometre-sized particles in panel (*a*), the minimum *q* value is already in the Porod region, *i.e.* 1/*q* is small compared with the particle size. Further complexity is added by the coherence length, which limits the area of coherent interaction and thus the sampling depth.

In a typical SANS experiment, interference-like patterns of nano-scaled particles are observed at low *q*, followed by an exponential drop at larger *q* above the constant incoherent background. In our study, no such distinct features are observed between *q* = 0.1 and 0.4 nm^−1^ in Fig. 3 ▸ and the SANS data show only an exponential Porod scattering behaviour. Above 0.4 nm^−1^ only background scattering is observed. This is because the graphite particles studied here (tens of micrometres) are large in comparison with 1/*q* and the lowest achievable *q* values are still too high (corresponding to 1/*q* of a few hundred nanometres) to resolve these features, as illustrated in Fig. 6 ▸. The *q* range in our experiments was limited to a single sample-to-detector distance in order to have the high time resolution required for *operando* SANS data at C-rates of C/5 and C/2. In other experiments where a larger *q* range could be measured, similar scattering features have been observed.

The SANS signals at the lowest *q* value start directly in the Porod region, where the particle size is of the order of 1/*q* or larger, so that the scattering cross section can be written as
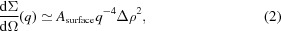
and is thus proportional to the object surface *A*_surface_ and Δρ^2^. The experimentally determined exponent of −3.8 (see discussion of Fig. 3 ▸) is close to the theoretical value of −4. The relative SANS contrast of material *A* in a matrix *B* can be described by the squared difference of scattering length densities which is the squared scattering contrast,
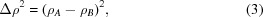
where ρ_*A*_ and ρ_*B*_ are the material-specific scattering length densities of materials *A* and *B*. The dependence of the macroscopic scattering cross section on Δρ^2^ was used earlier for simple particle scattering (Grillo, 2008 ▸) and more recently for scattering from micrometre-sized battery materials (Seidlmayer *et al.*, 2015 ▸; Sacci *et al.*, 2015 ▸). In Appendix *A* [App appa], a new and detailed mathematical derivation is given for the scattering from a multi-phase material with large particles, *i.e.* for typical Li-ion battery active materials as investigated here, which confirms the proportionality of the SANS scattering cross section (and also of the integrated intensity) to the squared difference in scattering length density Δρ^2^. This is also true for the general case and independent of the experimentally motivated Porod equation above.

A major difference from standard SANS data evaluation is that our sample volume cannot be seen as one matrix domain with nano-scaled inhomogeneities, because the dimensions of the graphite particles (and of most Li-ion battery active materials) are large in comparison with the neutron coherence length. In the SANS experiments shown here, the transverse coherence length given by *l*_coh_ = λ*L*/(4*d*_C_) is 120 nm, based on the used wavelength λ = 6 Å, the collimation length of *L* = 8 m and the collimation aperture diameter of *d*_C_ = 10 mm. This coherence length, which is illustrated in Fig. 6 ▸, is much smaller than the graphite particle size or the electrode thickness. Thus, the contributions of the electrodes or of particles far away from each other add up only incoherently. The volume of coherent interaction for which the scattering laws are valid is limited by the coherence length, so to get the overall cross section from the measured sample, we sum the scattering contributions from all coherence volumes in the sample. As a consequence, for the delithiated Li/graphite half-cell one must sum the independent contributions of the relevant interfaces in the cell, which are proportional to the local values of Δρ^2^ (with the appropriate constant, *c*_2_, which is in fact a function of *q* and *c*_0, other_ for the background, as shown in Appendix *A* [App appa]),
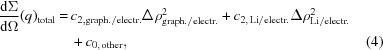
where the first term on the right-hand side refers to the graphite particle/electrolyte interface, the second term to the lithium surface/electrolyte interface and the last term to the contributions from the background [note that equation (4) corresponds to equation (18) in Appendix *A* [App appa] where we have inserted the explicit electrode names]. From this, the integrated intensity Γ can be written as
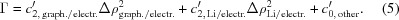
Here, we have only considered one interface between the graphite active material and the electrolyte (graph./electr.) and another one between the Li foil and the electrolyte (Li/electr.). This is a valid simplification because other interfaces do not contribute significantly to the overall scattering signal, as will be discussed by considering the theoretical Δρ^2^ values listed in Table 1 ▸. Furthermore, it should be noted that upon lithiation the graphite/electrolyte interface term in equation (5) will have to be replaced by a term describing the LiC_12_/electrolyte interface and, ultimately, by a term describing the LiC_6_/electrolyte interface. The separator and the binder polymer are chemically inert (at least during a few cycles) and do not change upon cycling.

Table 1 ▸ lists the scattering length density ρ for the different materials in our Li/graphite and Li/Li cells, as well as the squared difference Δρ^2^ (referred to as the squared scattering contrast) for the different material interfaces between any given phase *A* and an interfacing phase *B* (ρ values calculated with the software *SASfit*). The squared scattering contrast for the interface between the various graphite phases and the electrolyte is by far the largest, ranging from 26 to 40 × 10^20^ cm^−4^ (first three rows in Table 1 ▸). On the other hand, the squared scattering contrast for the interface between the various graphite phases is negligible, with values of <0.4 × 10^20^ cm^−4^ (second- and third-last rows in Table 1 ▸). Finally, the squared scattering contrast from the Li/electrolyte interface has an intermediate value, but its value of approximately 5 × 10^20^ cm^−4^ is still rather small compared with that of the graphite phases with the electrolyte. In the following, we will compare the scattering signal calculated from these values with the measured results.

From Table 1 ▸ and the Li/Li cell measurement, we can estimate that the contrast from (lithiated) graphite Li_*x*_C_6_ to the electrolyte is the major contribution to the SANS integrated intensity, as according to Table 1 ▸ all signal changes are proportional to the difference in Δρ^2^ upon lithiation. Table 1 ▸ shows a Δρ^2^ decrease of 19% when graphite changes from unlithiated graphite (C) to LiC_12_ (squared scattering contrast relative to largest value decreasing from 100 to 81%, see first and second rows of the last column in Table 1 ▸) and another Δρ^2^ decrease of 15% to LiC_6_ (see second and third rows of the last column in Table 1 ▸), equating to a total Δρ^2^ decrease of 34% upon the complete lithiation of graphite to LiC_6_. While a large drop in the SANS signal upon lithiation is indeed observed (see Fig. 5 ▸), the overall drop in the integrated intensity of the SANS signal is only approximately one-half of what would be predicted on the basis of the calculated Δρ^2^ decrease. This is due to the fact that, in this simple approximation, we have not considered the background signal and the effect of a finite coherence length.

However, a careful application of equation (5) should allow us to calculate the expected signal change in more detail. The parameters we had not considered in the above simple approximation are the factors *c*_2_′ and the background 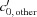, which contain information about the volume fractions and the detailed geometry of their respective contributions in relation to the overall sample. For this, one can first carefully evaluate equation (5) for the Li/graphite half-cell in which graphite is in its delithiated state, considering only the contributions from the Li/electrolyte and graphite/electrolyte interfaces, as well as additional information which is available for the three terms on the right-hand side of equation (5).

First, from other experiments (Seidlmayer *et al.*, 2015 ▸) we know that the inactive background contribution from the pouch, current collectors, separator and electrolyte alone is typically between 20 and 30% of the total scattering signal for a pouch cell. Because the cells used here and in the previous experiments were similar, we estimate a value of 30% for the background term 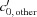. Second, from Fig. 4 ▸ one can see that the integrated intensity of the Li/Li cell on an absolute scale (∼2.4 × 10^−9^ nm^−2^) is approximately 40% of that of the Li/graphite cell (∼5.9 × 10^−9^ nm^−2^). After subtracting the background, we thus get a contribution of 10% for the Li/electrolyte term. Third, we attribute the remaining 60% to the graphite/electrolyte interface term. There is a small error because we do not account for the second Li interface, but we neglect it for the moment since the scattering contrast of the Li/electrolyte interface is in any case smaller than that of the graphite/electrolyte interface. Using the above estimates, the different terms in equation (5) can be approximated as follows:
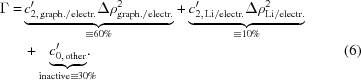
Here, we insert the measured integrated intensity of Γ = 5.9 × 10^20^ cm^−4^ in the delithiated graphite state and the squared scattering contrasts from Table 1 ▸, *i.e.* 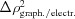 = 39.99 × 10^20^ cm^−4^ and 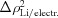 = 4.68 × 10^20^ cm^−4^, and then separately calculate the constants *c*_2_′.

For the graph./electr. interface,
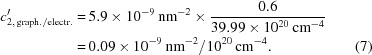
For the Li/electr. interface,
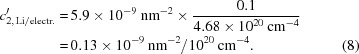
The integrated intensity for the graphite/Li half-cell with graphite in the fully lithiated state (LiC_6_) can now be calculated by inserting 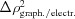 = 26.20 × 10^20^ cm^−4^,
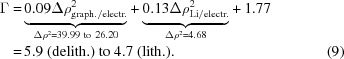
Here, Δρ^2^ and 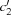 are given in the units above, so that the integrated intensity Γ is given in units of 10^−9^ nm^−2^. Thus, for the half-cell with fully lithiated graphite (*x* = 1, *i.e.* LiC_6_), one gets a total integrated intensity of 4.7 × 10^−9^ nm^−2^. This represents a drop of −21% in the integrated intensity Γ, which is reasonably close to the measured value of −15% in Fig. 5 ▸ (red curve). The integrated intensity drop to halfway at *x* = 0.5 (contrast of LiC_12_ particle to electrolyte) is projected to be −11%, which is again close to the experimentally observed −7%. The change in integrated intensity is reversible upon delithiation, except for a remaining absolute difference of 0.3 × 10^−9^ nm^−2^ at *x* = 0 after one C/5 cycle, which is probably the result of a changed background contribution from the Li anode (due to increased surface roughness caused by lithium plating).

### 4.1. Explaining the shape of the curve   

**Figure 7:**
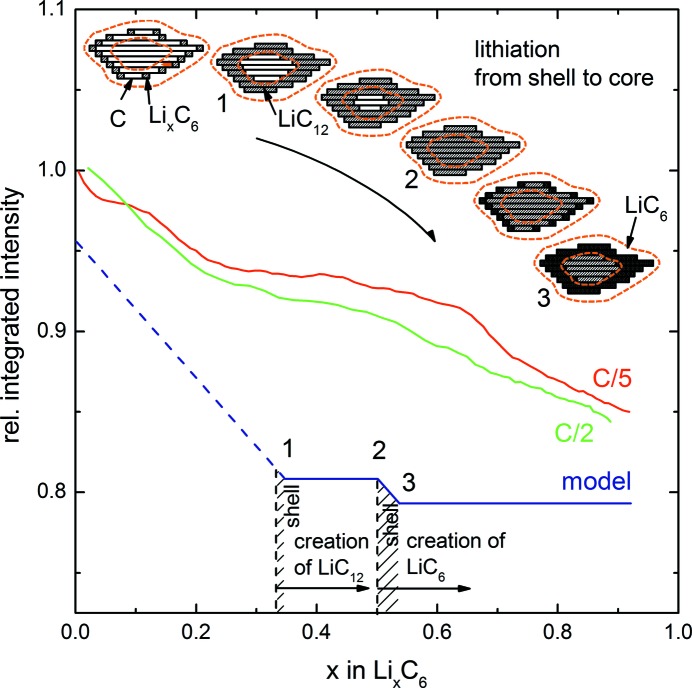
A scheme to explain the SANS data during lithiation of graphite. A comparison is shown of the smoothed relative integrated intensity normalized to the first value (red, green) with a simple model (blue) which is based on the creation of LiC_12_ and LiC_6_ in the particle shell defined by the coherence length (shaded area) and the interior. Points of interest are marked in accordance with the discussion. The curve proceeds from left to right during lithiation.

Above, it was shown how contrast changes determine scattering, but the limited area of coherent interaction has not been taken into account yet. In fact, two constraints apply: the limited coherence length, and the fact that only the interface between the active material and the electrolyte contributes significantly to scattering. Thus, the overall scattering cross section or integral intensity Γ only varies when the contrast changes within a surface shell of the active material particle (compare Fig. 6 ▸ with Fig. 9 in Appendix *A* [App appa]). This surface shell has the extension of one coherence length into the particle. In Figs. 7 ▸ and 8 ▸, the integrated intensity Γ is plotted in detail for lithiation and delithiation of graphite, with the data being normalized to the first integrated intensity value of the C/5 curve.

Fig. 7 ▸ (upper part) shows the lithiation of graphite in detail, if it were to proceed from the outside to the inside of the particle. For simplicity only two stages are shown. Upon lithiation LiC_12_ starts to build at the surface and the front propagates into the particle. When the particle is saturated with LiC_12_, LiC_6_ will start to propagate from the surface to the inside. Three distinct points can be identified where the shell contrast and integrated intensity change. Point 1 marks the full lithiation of the shell with LiC_12_; thereafter, the integrated intensity should stay constant since the shell remains unchanged. Point 2 marks the onset of LiC_6_ formation near the surface, which continues until point 3 which marks the completion of LiC_6_ in the shell, after which the integrated intensity remains unchanged again. A surface shell limited by a coherence length of 120 nm represents a share of 3% of the total volume of a 22 µm particle. Accounting for particle size variation, let us assume twice that figure, *i.e.* a roughly 6% share of shell volume. The 6% share is represented by the shaded areas in Figs. 7 ▸ and 8 ▸. From theory, point 1 is where the complete particle consists of LiC_18_ (*x* = 0.33) plus the 6% shell that is already LiC_12_, thus for point 1 we get *x* = 0.33 + (0.5 − 0.33) × 6%, *i.e.* *x* = 0.34. Point 2, where the whole particle is LiC_12_, lies at *x* = 0.5, and point 3, where the shell is filled up to LiC_6_, lies at *x* = 0.5 + (1 − 0.5) × 6%, *i.e.* *x* = 0.53. From the XRD experiment we see that the onset of LiC_12_ is earlier, already at *x* ≃ 0.2, which could be due to incomplete lithiation and other effects as discussed below.

**Figure 8:**
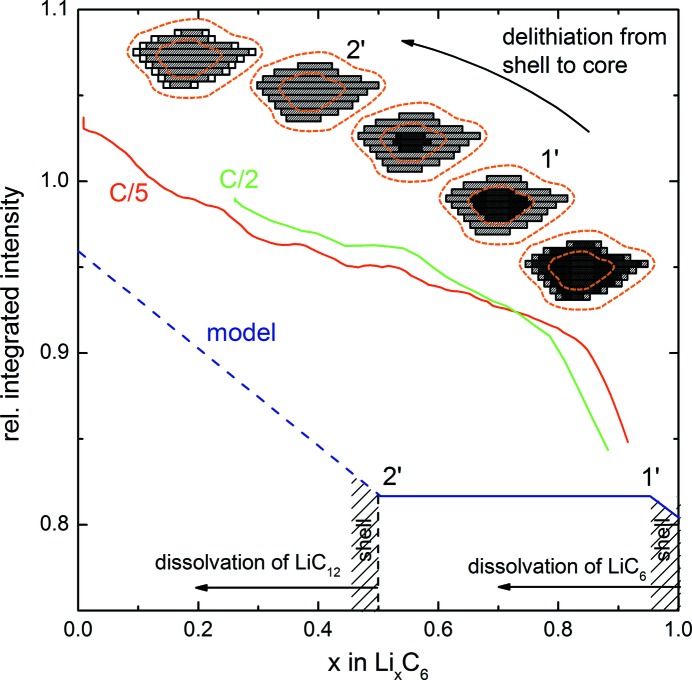
A model explaining the SANS data during delithiation of graphite. A comparison is shown of the smoothed relative integrated intensity normalized to the first value (red, green) with the model (blue) which displays the decrease in LiC_6_ and LiC_12_ in the particle shell given by the coherence length (shaded area) and interior. Points of interest are marked in accordance with the discussion. The curve proceeds from right to left during delithiation.

**Figure 9:**
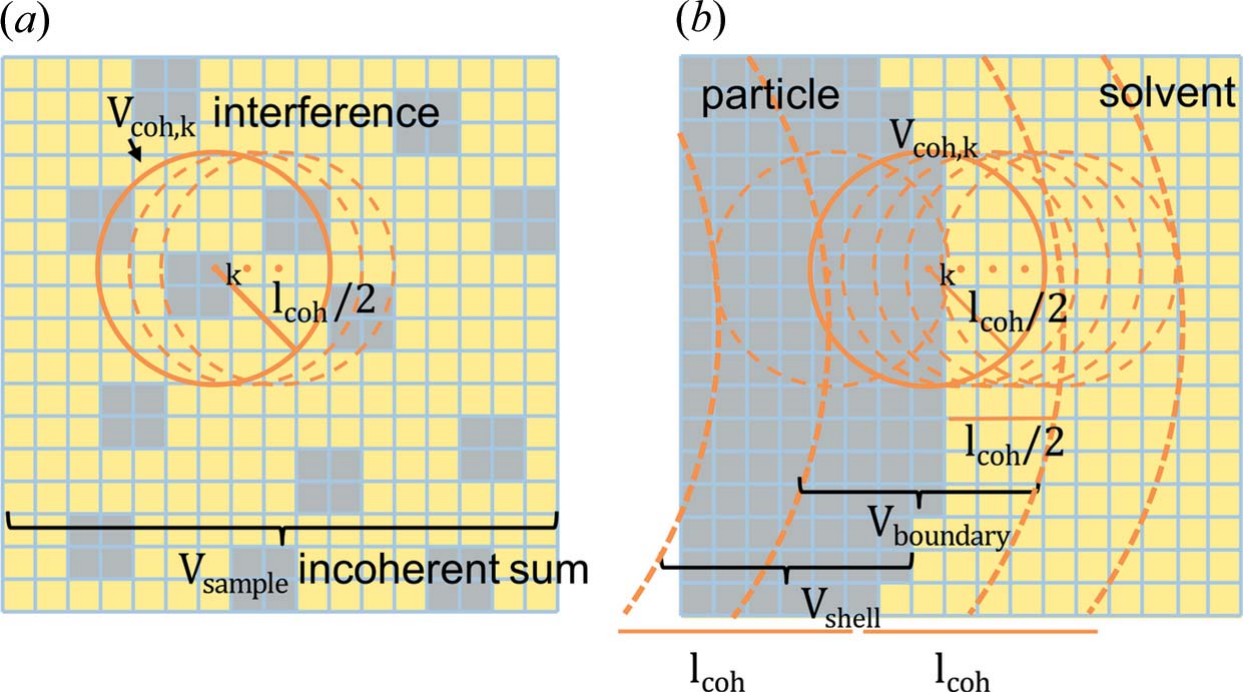
An illustration of the calculation mesh and the coherence volumes centred at each mesh element *k*, (*a*) for small particles and (*b*) for a large particle with two phases, *e.g.* active material and electrolyte or solvent. One square is of the order of 30 nm.

During delithiation, which is shown in detail in Fig. 8 ▸, we start from the fully lithiated particle and begin to delithiate the outer shell. The contrast varies until the shell has changed to LiC_12_ at point 1′. The next change in contrast at point 2′ occurs after the particle is completely transformed into LiC_12_ and more delithiation will create lower lithiated phases at the surface. From theory, point 1′ should lie at *x* = 0.97 (half the shell from 100%) and point 2′ at *x* = 0.5.

In Figs. 7 ▸ and 8 ▸, the shape of the model with a plateau in the SANS integrated intensity is reproduced by the experiment, even though the shape is somewhat distorted. Points 1 (when shifted to *x* = 0.2 as suggested by XRD) and 1′ agree fairly well with the experimental curves, but the other points are shifted slightly and the very simple model is not able to describe the experimental results in full.

### 4.2. Distortion of the curve and validity of assumptions   

The deviation of the experimentally observed integrated intensity from the simple core–shell model can be attributed to several effects. First, the size of the graphite particles in the sample is represented by a size distribution with a standard deviation of 11 µm around the mean particle size (by volume) of 22 µm. Thus, the ratio of coherence shell volume to particle volume varies substantially (6.5:3.2:2.2% for 11:22:33 µm) and the shape of the integrated intensity curve is smoothed out. Furthermore, incomplete lithiation in the inner volume of the particle would lead to an earlier onset of new phases at the surface during lithiation, resulting in a shift of the reference points to the left. From the XRD experiment we know that LiC_12_ forms already at *x* ≃ 0.2, which means that the particle is not fully lithiated up to LiC_18_ which would be at *x* ≃ 0.33. The incomplete lithiation can shift and smooth the curve, especially at high C-rates, as indeed observed in Fig. 5 ▸. Furthermore, the SANS data represent all particles in the electrode where homogeneity across the electrode dimension might vary, though at low rates this should be negligible. We have assumed stable phases that coexist in separated domains during cycling, which is confirmed by the XRD data which show distinct and coexistent phases within the cell under test for the chosen rates. Also, we have neglected volume changes of the phase geometry so far, but we have considered the density change for the scattering length density calculation. The volume change of graphite upon lithiation, which is up to 13% (Dolotko *et al.*, 2012 ▸), could indeed influence the SANS signal because the surface of all particles and thus the volume of the shell increases. However, the experimental data show no increase in SANS integrated intensity with lithiation due to surface growth, but instead a decrease in intensity which can only be explained by the discussed change in scattering length density contrast. The experimentally observed values for the change in the integrated intensity are, however, lower than expected from the contrast change alone. This observation might be explained by a superposition with the volume change effect.

## 5. Summary   

In the past, we had shown that the SANS integrated intensity signal from an NMC/graphite pouch cell varies with the state of charge, which we had hypothesized to be mostly due to changes upon graphite (de-)lithiation (Seidlmayer *et al.*, 2015 ▸). To confirm this hypothesis, we conducted analogous experiments with an Li/graphite half-cell, thereby removing any contribution of the NMC cathode. The signal changes are similar to those observed in our 2015 study (Seidlmayer *et al.*, 2015 ▸), which can now be attributed clearly to graphite lithiation. *Operando* XRD data obtained on the same half-cell confirm a homogeneous lithiation/delithiation across the entire electrode because the diffraction pattern shows only the subsequent evolution of one or at most two graphite stages at a time and not the coexistence of multiple stages. Based on a previously reported qualitative model to describe the SANS signal, we have developed a full theoretical model which explains the SANS integrated intensity signal from an Li-ion battery cell, showing that it is proportional to the squared difference in scattering length density.

For the Li/graphite cell examined here, the SANS integrated intensity signal stems mostly from the interface of the electrolyte phase with the (lithiated) graphite phase, and is restricted by the coherence length to a surface shell of the particle (*i.e.* to a shell thickness corresponding to the coherence length). This enables the SANS method to obtain information about the progress of lithiation across the graphite particles.

This model is applicable for materials where the particle sizes are (much) larger than the transverse coherence length of the experimental setup (120 nm in the experimental data discussed here). In the context of lithium-ion batteries, this means that it applies to many anode and cathode active materials (*e.g.* graphites, NCMs), while it does not apply to most conductive carbons. The measured overall difference of the SANS integrated intensity signal during cycling of the Li/graphite cell agrees to within 30% of the theoretically predicted values from contrast variation upon graphite lithiation. The observed signal shape with plateau-like features is explained qualitatively by the core–shell model with a finite coherence length. The SANS data analysis suggests that graphite stage coexistence evolves directly on a particle scale. This shell-to-core lithiation of graphite particles confirms the current view in the literature (Grimsmann *et al.*, 2018 ▸; Bauer *et al.*, 2017 ▸, 2016 ▸).

SANS, as a non-destructive measurement method, could also be used for other active materials such as Si in the future, where the understanding of the lithiation process is key to stable cycling. The compatibility of the method with thin pouch cells enables tests of materials in a realistic cell environment.

## Figures and Tables

**Table 1:** Scattering length density values and squared scattering contrast for the various material interfaces in an Li/graphite and an Li/Li cell The squared scattering contrast value is defined as Δρ^2^ = (ρ_*A*_ − ρ_*B*_)^2^. Phase density values were calculated from the crystallographic structure. Structure data for LiC_6_ and LiC_12_ were taken from Dolotko *et al.* (2012 ▸) and for graphite from Trucano & Chen (1975 ▸).

Phase *A*	Density of phase *A* (g cm^−3^)	Scattering length density ρ for phase *A* (10^10^ cm^−2^)	Phase *B*	Density of phase *B* (g cm^−3^)	Scattering length density ρ for phase *B* (10^10^ cm^−2^)	Squared scattering contrast of *A* relative to *B*, Δρ^2^ (10^20^ cm^−4^)	Squared scattering contrast relative to largest value (%)
C	2.26	7.5643	Electrolyte	1.15	1.2407	39.99	100
LiC_12_	2.23	6.9331	Electrolyte	1.15	1.2407	32.40	81
LiC_6_	2.20	6.3597	Electrolyte	1.15	1.2407	26.20	66
Polypropyl­ene (C_3_H_6_)_*n*_ (inner pouch, separator)	0.95	−0.3379	Electrolyte	1.15	1.2407	2.49	6
6-Nylon (C_6_H_11_NO) (outer pouch)	1.08	0.8025	Air	0.00	0.0043	0.64	2
C	2.26	7.5643	LiC_12_	2.23	6.9331	0.40	1
LiC_12_	2.23	6.9331	LiC_6_	2.20	6.3597	0.33	1
Li	0.56	−0.9232	Electrolyte	1.15	1.2407	4.68	12

## Appendix A Development of a theoretical model of SANS for battery materials

In general, SANS is used to study mesoscopic structures on the length scale of 1–300 nm. The usual application of SANS is *e.g.* to study nanoparticles in suspensions or precipitates in a solid matrix with such typical dimensions. SANS is well suited for an *operando* measurement because of the high neutron flux. Other small-angle neutron scattering techniques, such as very small angle neutron scattering and ultra-small-angle neutron scattering, which resolve even smaller *q* and have a longer coherence length, could be used to apply this measurement concept to even larger particles. Here, the SANS method is deployed for a mesoscopic system with layers up to 100 µm thick consisting partly of smaller active particles with dimensions in the range of tens of micrometres.

Starting from the well known SANS fundamentals, we can derive some simple relations. We start with the macroscopic scattering cross section dΣ/dΩ; this is proportional to the squared scattering amplitude *f*, which is the Fourier integral of the scattering length density ρ (Grillo, 2008 ▸; Kostorz, 1979 ▸; Frielinghaus, 2012 ▸). Usually, one integrates over the sample volume and normalizes to this volume, but here the sample volume cannot be seen as one domain because the dimensions of the battery materials are large compared with the neutron coherence length. We calculate the transverse coherence length as *l*_coh_ = λ*L*/(4*d*_C_) = 120 nm with a wavelength λ = 6 Å, a collimation length *L* of a few metres (*e.g.* 8 m) and a collimation aperture *d*_C_ = 10 mm in diameter.

The contributions of the anode, the cathode or particles far away from each other add up only incoherently. The volume of coherent interaction where the scattering laws are valid is limited by the coherence length, so to get the overall cross section from the system we sum (incoherently) the (coherent) scattering contributions from all coherence volumes *V*_coh_. This approach also follows the work of Majkrzak and co-workers who described a similar incoherent sum for neutron reflectometry with a small coherence length and large objects (Fitter *et al.*, 2006 ▸; Majkrzak *et al.*, 2006 ▸). To calculate this, we imagine a mesh (fine enough) with *N*_mesh_ elements where each element *k* is the centre of such a coherence volume. The dimensions of the mesh and the coherence volume are shown in 2D in Fig. 9 ▸, where particles of one phase (*e.g.* active material) are shown against a background phase (*e.g.* electrolyte). Note that for small enough particles [Fig. 9 ▸(*a*)] the integral is always approximately the same. For a larger particle [Fig. 9 ▸(*b*)], the integral can be very different, *e.g.* from a one-phase or two-phase region. We write down for the general case, and normalize by dividing by the sample volume *V*, following the literature [*e.g.* Grillo (2008 ▸) and Frielinghaus (2012 ▸)], and also by dividing by the number of mesh elements per coherence volume which is *V*_coh_/*V*_mesh_:

Here, ρ(**r**) = ρ_0_ + Δρ_*i*_ because we express the locally varying scattering length density ρ(**r**) as the contrast to a reference phase.

This means, in a multi-phase situation, several distinct phases *i* exist in the volumes *V_i_* where the scattering length density ρ_*i*_ is constant. The scattering contrast Δρ_*i*_ is thus defined as the difference of the scattering length density ρ_*i*_ of the phase from a constant non-contributing second phase (matrix or solvent) ρ_0_. Fig. 9 ▸(*b*) also shows that, for large particles, only those coherence volumes that lie within a boundary zone near the particle surface contain differences in ρ_*i*_, so that we can restrict the sum to all the mesh elements *N*_m. bound_ in this region, following Babinet’s principle. This principle states that only the relative contrast matters, so, for example, scattering from a solid sphere surrounded by air is similar to scattering from a spherical void of the same size in a solid bulk. This is true if the particle is one phase and the surrounding electrolyte the other, but we will see that we can assume the same for particles with several lithiated phases because the difference in ρ (and accordingly in Δρ) between *e.g.* C and LiC_6_ is very small. Following the multi-phase literature (Frielinghaus, 2012 ▸) we write

The integral  is a *q*-dependent scattering function which is integrated over the volumes of the phases containing all the geometric information,

The brackets indicate the statistically averaged value which is of course restricted to the coherence volume, and again for the sum *i* = *j* is allowed, while for *i* ≠ *j* we have to take the real part of  because of the change in the order of the integrals. The incoherent background is represented by the constant *c*_0_. We can simplify further by fixing the total number of particles *N*_P_ and the (average) boundary volume *V*_bound_ per particle. For large particles *V*_bound_ is roughly the volume of the shell *V*_shell_ which lies within one coherence length of the surface. We insert *V*_mesh_ = *V*_bound_/*N*_m. bound_ *N*_P_ = *V*_shell_/*N*_m. bound_ *N*_P_,

Again, *c*_0_ represents the incoherent background and *c*_1_ is a constant factor accounting for systematic errors in the sum, *e.g.* an asymmetric coherence volume (longitudinal, transverse) and overlapping coherence volumes from neighbouring particles. Now, the scattering cross section is a function of squared and mixed Δρ contributions, and the phase volumes times the scattering function . The function is evaluated and averaged over the coherence volumes of the boundary zone. When we assume that there is only a single-phase situation (*i.e.* one phase of an active particle to the electrolyte matrix background) we obtain the following expression for the scattering cross section, where we take the average in the second step:

The result is again a function that depends on the squared difference in scattering length density and the average of the squared phase volume times the *q*- and volume-dependent scattering function . We drop the directional property of *q* here because we deal with the assumption of isotropic scattering.

We outline the following situation. Imagine a single-phase situation (*e.g.* a single solid phase with Δρ versus electrolyte) where the scattering contributions are given by the average of Δρ^2^ weighted by the squared phase volume and the scattering function  representing the geometry. Now, when a second phase is created, we expect the transition to the two-solid-phase situation (plus electrolyte which is the matrix and thus the third phase) to be a smooth function, weighting the scattering length density differences by the respective phase volume fractions in all *V*_coh_. Finally, if all of the first phase transforms to the second, we are at the single-phase situation again and the averaged term, *i.e.* the squared volume times the *q*-dependent function , is exactly the same because the phase geometry is the same. In conclusion, the only difference between single phases occupying a similar volume lies in Δρ^2^. The dependence of the scattering intensity on Δρ^2^ is in line with the findings of Sacci *et al.* (2015 ▸), although they did not consider the effect of coherence length.

The last step to get the total macroscopic scattering of the battery is to sum the contributions of all the components in the battery. Because the pouch foil, separator, Cu and Al foils, electrolyte, and active materials are well separated on the length scale of the coherence length, we can add up these contributions incoherently. We write the scattering cross section as a superposition of contributions from the relevant interfaces,

When individual particles cannot be identified, the factor *c*_1_(*V*_shell_/*V*_coh_)*N*_P_ reduces to *V*_shell_/*V*_coh_, where the shell volume is given by the coherence length reaching into the actual structure. The relative weight of the summands is thus determined by the volume share (via *V*_shell_ *N*_P_), the surface area (via *V*_shell_) and Δρ^2^. As before, we restrict the multi-phase behaviour to only two phases at the interface, *e.g.* one solid phase and electrolyte. So we can calculate the start, intermediate and end points of the battery system, *i.e.* Li foil and graphite particles which are lithiated homogeneously in the respective shell volume. We assume that the active material to electrolyte interfaces are dominant and combine all other contributions (including incoherent background from electrodes and other materials) into the constant *c*_0, other_, so that we arrive at

In this equation, *c*_1_, *V*_shell_, *N*_P_, *V*_1_ and  are specific to the indicated interfaces, but for simplicity individual indices have been omitted. We can simplify further by defining the factor

For anode/electrolyte (a/e) and cathode/electrolyte (c/e) we arrive at

Now all the geometric information is contained in the factors *c*_2_ and this information is similar for any phase occupying the same volume (assuming the shape is unchanged). So, from this we could already calculate the difference from the lithiated to the delithiated state. In order to get an integral measure of the scattering we can either use the Porod invariant or use the simple integrated intensity of the scattering cross section over *q*. With the same reasoning as above, we obtain for the integrated intensity Γ

where again  collects all the background (now with the integral over *q*, denoted by the prime). We can simplify further by defining the factor ,

This leads to

Thus, we can compute the difference in integrated intensity Γ for a transition from the charged-state phase to the discharged solid state simply by

In the half-cell, the index anode/electrolyte is equal to Li/electrolyte and cathode/electrolyte is equal to graphite/electrolyte. We do not have to know the microscopic structure to compute  *ab initio* when we want to get the difference in integrated intensity ΔΓ, which is just proportional to the weighted difference in Δρ^2^ on the scale of the coherence length. The geometric factor  can be fitted to experimental data or derived from additional experiments with the individual materials.

In summary, we have confirmed the hypothesis that the integrated intensity scattering signal is proportional to the difference in Δρ^2^. The material contributions are weighted by the amount of shell volume at the phase interface or surface where contrast is generated.
